# Social Exclusion: More Important to Human Females Than Males

**DOI:** 10.1371/journal.pone.0055851

**Published:** 2013-02-06

**Authors:** Joyce F. Benenson, Henry Markovits, Brittney Hultgren, Tuyet Nguyen, Grace Bullock, Richard Wrangham

**Affiliations:** 1 Department of Human Evolutionary Biology, Harvard University, Cambridge, Massachusetts, United States of America; 2 Department of Psychology, Emmanuel College, Boston, Massachusetts, United States of America; 3 Département de Psychologie, Université du Québec à Montréal, Montreal, Quebec, Canada; University of Milan, Italy

## Abstract

Theoretical models based on primate evidence suggest that social structure determines the costs and benefits of particular aggressive strategies. In humans, males more than females interact in groups of unrelated same-sex peers, and larger group size predicts success in inter-group contests. In marked contrast, human females form isolated one-on-one relationships with fewer instrumental benefits, so social exclusion constitutes a more useful strategy. If this model is accurate, then human social exclusion should be utilized by females more than males and females should be more sensitive to its occurrence. Here we present four studies supporting this model. In Study 1, using a computerized game with fictitious opponents, we demonstrate that females are more willing than males to socially exclude a temporary ally. In Study 2, females report more actual incidents of social exclusion than males do. In Study 3, females perceive cues revealing social exclusion more rapidly than males do. Finally, in Study 4, females’ heart rate increases more than males’ in response to social exclusion. Together, results indicate that social exclusion is a strategy well-tailored to human females’ social structure.

## Introduction

Thousands of studies on sex differences in human aggression across cultures and age intervals have divided aggression into direct versus indirect forms, then provided post hoc evolutionary, biosocial, and socialization explanations [1]–[6]. Research with non-human primates provides a different starting point, one in which sex differences in social structure form a theoretical basis for predictions. Specifically, research has established that human males interact in larger, more interconnected groups with unrelated same-sex peers, while females prefer isolated one-on-one interactions [7]–[9], a difference that appears in a nascent form in infancy [10] and has been linked with infant levels of testosterone [11]. Further, unrelated human males provide more instrumental assistance to one another than unrelated human females do [12]–[14]. One of humans’ two closest living genetic relatives, chimpanzees (*Pan troglodytes*), exhibits the same sex difference in social structure and instrumental benefits [15], [16], facilitating predictions regarding the relation between social structure and patterns of aggression.

Across chimpanzee communities, males compete for dominant positions within a group but maintain the integrity of the group to ensure victory over hostile neighboring groups [17]. Further, within a community, males engage in more cooperative activities with one another than females do [18]–[20]. In contrast, adult female chimpanzees associate primarily with their offspring and otherwise remain solitary. Top-ranked females occasionally form temporary coalitions in order to oust a newcomer female or hurt a low-ranked community female [21], [22]. Social exclusion of lone females from inside or outside the community reduces pressure on resident females to share scarce food resources or territories that provide protection from hostile groups [23]–[25].

From a theoretical perspective, in species in which one sex interacts as a group and the other does not, social exclusion produces differential benefits and costs (Benenson, 2009). For human males, direct intra-community competition [2] co-exists with an abundance of intra-group cooperative activities including inter-group warfare in which larger group size promotes victory [12], [13], [26], [27]. Large groups of unrelated females do not provide a similar benefit to human females. For human males, use of social exclusion reduces intra-individual competition, but simultaneously weakens the group in inter-group contests. Thus competing for dominance within a group while maintaining group integrity would constitute a more optimal strategy than social exclusion. In contrast, for human females, social exclusion leading to elimination of vulnerable females should enhance resident females’ access to resources, such as food and prime territory, as well as increasing available assistance from kin and sexual partners.

In humans, social rejection’s impact is comparable to physical pain for both sexes [28], [29]. However, it is unclear whether social exclusion hurts one sex more than the other [30]. For example, in response to social exclusion, some studies find greater cortisol concentrations in women versus men [31], while others do not find sex differences in cortisol concentrations [32] or find blunted cortisol responsiveness in women compared to men [33]. Likewise, some studies find that social exclusion depresses affect more in women than men [33], while other studies find similar levels of negative affect in the two sexes [32]. Nonetheless, recent research shows that when individuals are threatened directly by social exclusion, females’ behavioral reactions are stronger than males’ reactions [34]. Why should this be?

Our theoretical analysis suggests that because human females do not benefit as much as males from group membership, social exclusion should be used more frequently as an aggressive strategy by females and should correspondingly be experienced more frequently by females than males. Surprisingly, to our knowledge, no study has examined this. While some anecdotal evidence indicates use of social exclusion in adolescent girls [35], [36], boys have not been included in these studies. Existing empirical studies of social exclusion have been embedded within measures of indirect aggression, including relational aggression [37], social aggression [38], and covert aggression [3]. However, social exclusion need not occur indirectly, nor are there any theoretical reasons that it should be related to other measures of indirect aggression. Objective indices of social exclusion are difficult to collect because extensive interviews indicate that individuals may not be conscious of committing acts of social exclusion [39].

The following studies consequently were designed to investigate two hypotheses: 1) that females utilize social exclusion more than males do and 2) that females exhibit greater perceptual sensitivity than males to social exclusion.

### Ethics Statement

All studies were approved by the IRB of Université du Québec à Montréal or Emmanuel College. Consent was obtained on the first page of the computer program for studies using computerized games, by completing the questionnaire for single page frequency studies, or by signing a consent form for the heart rate study.

## Study 1

We constructed an experimental paradigm which directly modeled exclusionary alliances in non-human primates, by adapting a computerized game [40]. We revised the game to model temporary coalition formation in which the sole purpose was to oust a third party, after which the coalition disintegrated and the partners competed against one another. This type of purely exclusionary alliance is formed by some non-human primates [41].

In the game, a participant competed against 2 fictitious opponents. The participant could either compete individually to gain a reward- or form a temporary coalition simply to defeat the third party, after which the coalition partners would compete against one another for the indivisible reward. If a participant chose to form a temporary coalition with one opponent, the coalition would pool their strength to compete against the lone opponent. If the lone opponent was eliminated, then the coalition partners would compete against one another to determine who would win all the points. Thus, the sole result of a temporary coalition was to increase the chances of eliminating a competitor. Importantly, the model was designed so that, irrespective of participants’ choices, their individual chances of success were identical.

### Method – Study 1

#### Participants

80 university students from Montreal, Canada (40 females, Age: *M* = 23.6 years, *SD* = 9.75; 40 males, Age: *M* = 25.8 years, *SD* = 5.10) individually competed for money against two fictional same-sex opponents in a computerized game.

#### Procedure and material

On each of 28 rounds, the player’s “strength” (the probability of winning all points in a round when competing individually) varied randomly from 20–80% (in 10% increments) with 4 rounds at each level of strength. In each round, the player’s plus the opponents’ strength equaled 100%, with the two opponents’ strength unequally apportioned. Instructions indicated that only the player earning the highest number of points would win up to $5. The players were told how much they had won only when the game ended.

On each round the player was informed of his strength and that of his or her opponents, and was given three choices: (1) compete alone, with strength indicating probability of winning all points; (2) form a coalition with either opponent, with strengths summed, and if the coalition won, members would subsequently have to compete to determine who would win all the points; or (3) form a coalition with both opponents, with coalition members then competing amongst each other immediately to determine who would win all the points. Probabilities and payoffs were clearly demonstrated before the game. Although each choice produced the same probability of winning, players were not told this explicitly. Choosing to form a temporary coalition with one other opponent (exclusionary coalition) simply increased the chances of eliminating the lone opponent without altering the participant’s probabilities of winning. The third choice simply deferred competition one additional turn. We included this option in order to control for the possibility that one sex might be intuitively attracted by such deferral.

### Results and Discussion – Study 1

**Figure 1:**
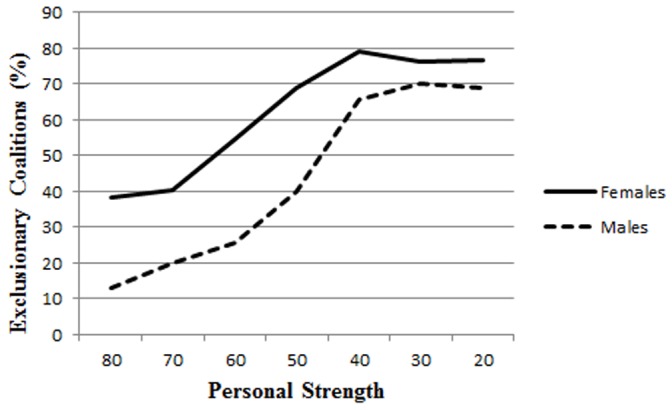
Percentage of exclusionary coalitions at each strength level (probability of winning) for females and males.

We calculated the number of times (out of 4 choices) that participants chose to form an exclusionary coalition with one opponent for each level of strength. A repeated measures analysis of variance (ANOVA) on percentage of exclusionary coalitions at each strength level, with strength as a between-subjects variable and sex as an independent variable showed significant effects of strength level, *F*(6, 73) = 16.61, *p*<.0001, and sex, *F*(1, 80) = 9.99, *p*<.02. Both females and males formed exclusionary coalitions more often when they were weaker. As predicted, out of 28 trials, females (*M* = 17.30, *SD* = 8.06) formed more temporary exclusionary coalitions than males (*M* = 12.13, *SD* = 6.49) (see Figure 1).

We then examined number of choices to form a coalition with both opponents (deferred competition). A repeated measures analysis of variance (ANOVA) on number of choices to defer competition at each strength level, with strength as the between-subjects factor and sex as the independent variables showed only a significant effect of strength level, *F*(6, 73) = 4.24, p<.001, with individuals choosing this strategy only at the lowest levels of strength. Over the 28 trials, females (*M* = 3.88, *SD* = 7.95) and males (*M* = 3.30, *SD* = 7.47) chose this strategy equally often.

Prior studies using this computerized game demonstrated that when rewards were shared between partners, no sex differences appeared in strategic decisions [40], unless players are under a direct, explicit threat of being the target of social exclusion [34]. The present study used a game in which rewards were not shared, and players were not under any exclusionary threat. In this context, the only consequence of forming a coalition with one opponent was to increase the chances of eliminating the third competitor, as occurs in some primate alliances [41]. Results clearly show that women are more likely than men to form these exclusionary coalitions.

## Study 2

In order to validate these experimental results using a more naturalistic measure, we simply surveyed a random sample of male and female university students to tabulate the frequencies with which they had been targets of social exclusion.

### Method – Study 2

#### Participants

74 university students from Montreal, Canada (37 females, Age: *M* = 25.2 years, *SD* = 7.96; 37 males, Age: *M* = 25.6 years, *SD* = 5.23) participated in this study.

#### Procedure and materials

Participants completed a single page questionnaire individually in a library. The questionnaire defined social exclusion as an episode in which same-sex friends or close acquaintances took part in a joint activity without the participant, in a situation where the participant was available and would have expected to be included. Each participant was asked to list any episodes in which they had been the target of such social exclusion in the past year and to write a brief description of each.

### Results and Discussion – Study 2

Consistent with the computerized game, females (*M* = 1.92, *SD* = 1.12) reported significantly more episodes of social exclusion than males (*M* = 1.30, *SD* = 1.08) did, *t*(72) = 2.44, *p*<.02. Further, only 2/37 females (5%) compared with 11/37 males (30%) reported never having been socially excluded in the past year, *X^2^*(1) = 7.56, *p*<.01.

## Study 2a

Although the results of study 2 are consistent with our hypothesis, they might be due to a differential ability to remember social information. In order to examine this possibility, we asked a sample of men and women to report on the number of times that they had received significant help from a same-sex peer. We chose this social event, because we expected it to occur relatively rarely, but we did not have any theoretical basis for expecting the sexes to differ.

### Method – Study 2a

#### Participants

70 university students from Montreal, Canada (35 females, Age: *M* = 23.9 years, *SD* = 5.99; 35 males, Age: *M* = 21.4 years, *SD* = 4.85) participated in this study.

#### Procedure and materials

Participants completed a single page questionnaire individually in a library. The questionnaire defined episodes of significant aid as an episode in which same-sex friends or close acquaintances helped the participant to accomplish something that they would not have been able to do by themselves. Each participant was asked to list any episodes in which they had been the target of such assistance in the past year and to write a brief description of each.

### Results and Discussion – Study 2a

In contrast to the results of Study 2, both females (*M* = 2.4, *SD* = 1.60) and males (*M* = 2.71, *SD* = 1.27), reported very similar rates of being helped by a same-sex peer, *t*(68) <1.

The results of this study suggest that females’ reporting of greater frequency of exclusion is not due to any general sex difference in recall of social information. Together, these studies suggest that compared to males, females utilize social exclusion with peers more frequently.

This has consequences not only for behavior and emotional reactions, but also for the way that social information is processed. Past research demonstrates that males and females differentially process social information with varying efficiency that reflects their respective social structures, with females focusing more on individuals and males on groups [42], [43]. Information relevant to social dynamics that is uniquely salient to one sex generates more arousal and hence is processed more attentively by that sex.

Social exclusion is particularly painful [28]. Thus, the greater frequency of social exclusion among females suggests that females should be strongly primed to attend to and process information about social exclusion. We examined this hypothesis in the following two studies.

## Study 3

In Study 3, we investigated this hypothesis using a novel paradigm developed to examine social rule extraction [44]. This method involves presenting computerized interactions between cartoon characters in which precise cues predict the occurrence of social exclusion. Females should detect cues predictive of being a potential target of social exclusion more rapidly, because they would have more experience with the consequences of exclusion.

In this method, each scene depicts a silent interaction in which the participant takes the role of an avatar approaching a group of human-looking avatars. The participant views two members of the group interacting, and in each scene one of two types of movement is always included: either an avatar uses a cell phone or an avatar shakes hands with another avatar. Whenever a cell phone is used, the participant’s avatar is always included in the subsequent interaction. Whenever two avatars shake hands, the participant’s avatar is always excluded from further interactions. Each scene varies across several dimensions (such as type of clothes, other movements, position), which are counterbalanced equally across scenarios that include using a cell phone or shaking hands. In a prior study of social rules, males were shown to be more efficient than females at identifying social cues using this method (Markovits, unpublished data). The hypothesis here however is that females should be more efficient than males at learning to extract relevant cues to predict social exclusion.

### Method – Study 3

#### Participants

62 university students from Montreal, Canada (31 females, Age: *M* = 22.4 years, *SD* = 10.62; 31 males, Age: *M* = 24.9 years, *SD* = 8.55) participated.

#### Procedure and materials

The experiment was conducted individually on a portable computer using a Visual Basic 6 program constructed for this purpose. Females viewed interactions with female avatars, while males viewed interactions with male avatars. Participants were presented with instructions that stated (translated from the original French):

“In what follows, we are going to ask you to pretend to be in a strange place where everything happens in a different fashion from what you're used to. You are going to watch interactions in which you are walking by yourself. You see some other people who you know well on the other side of the street. They are speaking to each other. You come closer to see what is happening. Later, you see one of these people is going into a house that the other people that you saw entered. Sometimes you are invited inside to join them, but sometimes you are not invited inside. Your task will be to discover how you can anticipate the reaction of these people.” Following these instructions, participants were first shown an interaction with a positive outcome, in which the participant is invited into the house, and then an interaction with a negative outcome, in which the participant is not invited inside.

Next, a participant received up to 20 interactions with each lasting approximately 20 s. For each interaction, the participant was shown all of the interaction except the final outcome (inclusion or exclusion), and they were asked to predict the outcome. For each prediction, the participant was informed whether his or prediction was correct, after which the participant was asked whether s/he had discovered a predictive rule. If a participant indicated discovery of a rule and subsequently provided four consecutive correct predictions, then the study ended.

Rules were categorized as accurate if they mentioned either the use of the cell phone as a cue to inclusion, shaking hands as a cue to exclusion, or both. Efficiency of rule discovery was defined by how rapidly the participant discovered the correct rule. When a participant indicated that s/he knew the rule and subsequently succeeded on 4 correct trials, the discovery trial was the number of the trial at when they indicated that they knew the rule; otherwise the discovery trial was set to 21.

### Results and Discussion – Study 3

We performed an ordinal logistic analysis with number of the discovery trial as the dependent variable and sex as the independent variable. This showed a significant effect of sex, *X^2^*(1) = 3.94, *p*<.05. The discovery trial was significantly lower for females (*M* = 10.2, *SD* = 5.63) than for males (*M* = 13.8, *SD* = 5.53), indicating that females were faster than males in identifying cues related to social exclusion. This study is thus consistent with the prediction that females’ heightened arousal to information about social exclusion allows them to detect cues predictive of social exclusion in a virtual environment faster than males.

## Study 3a

To ensure that the results were not due either to the specific cues used in this study or to any general sex difference in processing social information, the previous study was repeated with the participant’s avatar being a member of a group and thus not subject to individual exclusion. Parameters were otherwise identical to those of Study 3.

### Method – Study 3a

#### Participants

48 university students from Montreal, Canada (24 females, Age: *M* = 21.2 years, *SD* = 8.12; 24 males, Age: *M* = 23.6 years, *SD* = 9.09) participated.

#### Procedure and materials

The procedure and materials were identical to those of Study 3 with one exception. Participants were described as being part of a group of friends, with the outcome (exclusion, inclusion) directed towards the group.

### Results and Discussion – Study 3a

As before, we performed an ordinal logistic analysis with number of the discovery trial as the dependent variable and sex as the independent variable. This showed no effect of sex, *X* ^2^(1, N = 48) <1. There was no difference in the mean discovery trial between females (*M* = 10.9, *SD* = 5.62) and males (*M* = 11.3, *SD* = 6.08).

Once again however, the standardization provided by a computer simulation must be balanced by more ecologically valid evidence. Consequently, we conducted focus groups with a number of undergraduates to determine what types of aggression they commonly encounter. We then asked participants in a final study to imagine that they had just been involved in three types of frequently occurring aggressive incidents.

## Study 4

In Study 4, we asked participants to read about three common aggressive incidents, one of which included social exclusion, and describe their reactions to each while their heart rate was monitored continuously. Research demonstrates that the extent of arousal when processing information with emotional valence is related to the efficiency of storage and ease of recall (e.g. [45]. We thus predicted that females and males should show equally strong and negative subjective reactions to social exclusion as shown in numerous past studies [30], but that females would have a proportionally greater state of arousal than males to the social exclusion situation.

### Method – Study 4

#### Participants

20 females (*M*_age_ = 19.40, *SD* = 0.94) and 20 males (*M*_age_ = 19.35, *SD* = 1.42) between 18–23 years from a small college in Boston, MA participated.

#### Procedure and materials

Upon arrival at the laboratory, the experimenter attached electrodes to the participant’s two ankles and non-dominant wrist for continuous heart rate recording, and then left the room for 5 minutes during which the participant relaxed. Following that, the participant read 3 detailed descriptions of common aggressive incidents: social exclusion, physical aggression, and bystander aggression. For the social exclusion incident, the participant had to imagine not being invited to a New Year’s Eve party by a close same-sex friend, even after the participant has asked the friend what s/he was doing for New Year’ Eve. Afterwards, a mutual friend told the participant about the party. For the physical aggression incident, the participant had to imagine having an argument with a close same-sex friend at a party and the friend’s punching the participant causing a bloody nose which required stitches. For the bystander incident, the participant imagined his or her path being obstructed on the street by two same-sex individuals arguing loudly in a foreign language.

After reading about an incident and imagining it had just happened, the participant was given up to 5 minutes to write a description of his/her emotional reactions. Afterwards, an experimenter entered the room and asked the participant to complete 2 subjective evaluations on 5-point scales measuring how long it would take for the participant to recover from the incident (1 = immediately to 5 = never) and how angry the participant would feel (1 = not angry to 5 = very angry). To ensure privacy, the participant was alone in the room, and all written descriptions and scales were deposited in a slit in a sealed box. After the participant completed the evaluations, s/he was given 2 minutes to relax before proceeding to read about the next incident. Two sequences of incidents were utilized equally with females and males with the bystander incident always in the middle. Heart rate was analyzed for 3 minutes starting when participant began reading about the aggressive event.

### Results and Discussion – Study 4

To correct for individual reactions to aggression, all measures were divided by the corresponding response to the bystander situation. Subjective evaluations of duration of distress to social exclusion and physical aggression therefore were each divided by subjective evaluation of duration of distress to the bystander aggression incident. Likewise, subjective evaluations of anger to social exclusion and physical aggression were each divided by subjective evaluation of anger to the bystander aggression incident. Similarly, heart rate in response to social exclusion and physical aggression were each divided by heart rate in response to bystander aggression. Since age was significantly correlated with both subjective evaluations and heart rate across the entire time interval, we entered age as a covariate in all analyses.

Separate repeated measures ANOVAs on the corrected subjective evaluations of duration of distress and degree of anger with sex as the independent variable and age as a covariate yielded no significant effects. Female and male participants predicted that they would feel similarly both in terms of the time they would take to recover and in how angry they would feel when imagining confronting social exclusion and physical aggression (see Table 1).

**Table 1:** Corrected measures of predicted duration of distress, anger and heart rate as a function of type of aggression (social exclusion, physical aggression).

	Social Exclusion		Physical Aggression	
	*M*	*(SD)*	*M*	*(SD)*
Duration of Distress				
Females	2.25	(.65)	2.90	(.91)
Males	2.35	(.81)	2.90	(.97)
Degree of Anger				
Females	2.92	(1.03)	3.53	(1.44)
Males	2.94	(1.43)	3.17	(1.40)
Heart Rate				
Females	1.06	(.15)	1.01	(.09)
Males	0.97	(.12)	1.01	(.11)

In contrast, a repeated measures ANOVA on the corrected social exclusion and physical aggression heart rate measures with sex as the independent variable and age as a covariate yielded significant effects of type of aggression, *F* (1, 37) = 8.48, *p* = .006, which was qualified by significant interactions between type of aggression X sex, *F* (1, 37) = 4.60, *p*<.04, and type of aggression X age, *F* (1, 37) = 8.42, *p* = .006. Older participants exhibited lower heart rates than younger participants in response to the social exclusion incident. Tukey’s test, *p*<.05, showed that across ages, females’ heart rate was significantly higher than males’ in response to the social exclusion incident, but not in response to the physical aggression incident (see Table 1).

When the analysis was repeated with age as a between subjects factor (with participants divided by age into 18–19 years versus 20 years or older), the results were similar. A repeated measures ANOVA on heart rate with type of aggression as the repeated factor, and sex and age as the independent variables, yielded significant effects of type of aggression X sex, *F* (1, 36) = 8.32, *p* = .007, and type of aggression X age, *F* (1, 36) = 10.28, *p* = .003. Again, Tukey’s tests, *p*<.05, demonstrated that females’ heart rate was significantly higher than males’ heart rate in response to social exclusion, but no sex difference in heart rate appeared in response to physical aggression. Further, Tukey’s tests demonstrated that older participants (*M* = .95, *SD* = .16, *n* = 13) demonstrated significantly lower heart rate in response to social exclusion than younger participants (*M* = 1.04, *SD* = .13, *n* = 27) and than older participants (*M* = 1.03, *SD* = .11, *n* = 13) and younger participants (*M* = 1.00, *SD* = .09, *n* = 27) in response to physical aggression, none of whom differed from one another.

Consistent with the hypothesis, females show a stronger rate of arousal than males when asked to process information about a situation of social exclusion, despite similar levels of conscious distress. Importantly, this result occurred only during the first 3 minutes after the presentation of the aggressive incident. During the subsequent 2 minutes, the sex difference disappeared.

In summary, using two different perceptual measures, the results demonstrate that compared to males, females process information related to social exclusion with heightened attention and arousal.

## General Discussion

Past studies of social exclusion uniformly conclude that it is comparable in pain to physical injury [28], [29]. While generally the two sexes report similar levels of distress in response to social exclusion [30], when sex differences are found, females report more distress than males [33]. Our two initial studies utilizing a computerized model as well as a self-report measure provide evidence that human females confront social exclusion more frequently than males do. Our latter two studies demonstrate that females are more cognitively and perceptually sensitive than males to incidents of social exclusion.

The results of this series of studies thus are consistent with the theoretical model derived from non-human primates that suggests that human females confront social exclusion by same-sex individuals more frequently than males do [46]. Use of social exclusion likely provides benefits to human females by reducing survival and reproductive costs since fewer individuals compete for the same resources, including food, territory, and assistance from sexual partners. Benefits from female alliance formation may be limited because unrelated human females provide less instrumental help to one another than human males do [12], [14]. In contrast, human males who also can profit from reducing competition for mates and resources must balance these benefits against costs imposed by potential defeat by larger external hostile groups due to loss of intra-group allies. This suggests that human males must negotiate a compromise between the individual quest to attain dominance within the community and the individual’s need for intra-group alliances, especially during inter-group contests [17], [26], [47].

Traditional research on sex differences in non-human competition and aggression extrapolates from an animal model based primarily on male competition for mates [48]. Newer research suggests that non-human females also benefit from competing for resources, territory, breeding opportunities, and assistance with rearing offspring [49], [50]. Human models need to incorporate female competition as well. The formation of temporary exclusionary coalitions provides an elegant means by which females, either directly or indirectly, can minimize competition without incurring large costs.

How early experience with specific social structures translates into differential processing of social information deserves further study. Beginning in childhood, human females without the support of a group may be more vulnerable to social exclusion, providing an impetus for females to expend resources to identify an ally who may not provide other benefits [51]. Lone males may be less vulnerable to exclusion from the group, thereby alleviating pressure to invest in a relationship that may not otherwise be beneficial. In adulthood, males may be more able than females to use the threat of social exclusion to induce females to comply with their sexual demands [52], just as females may employ their sexuality to obtain benefits from males [53]. Understanding the interplay between females’ and males’ early experiences in differing forms of social structures and perceptual thresholds for social exclusion may illuminate sexually dimorphic motivations underlying human social bonds.
